# Adjuvant use of melatonin for pain management in dysmenorrhea — a randomized double-blinded, placebo-controlled trial

**DOI:** 10.1007/s00228-021-03234-6

**Published:** 2021-10-20

**Authors:** Lisa Söderman, Måns Edlund, Ylva Böttiger, Lena Marions

**Affiliations:** 1 Dept of Clinical Science and Education, Södersjukhuset, Karolinska Institutet, Stockholm, Sweden; 2 KBH, Karolinska Institutet, Stockholm, Sweden; 3 Dept of Biomedical and Clinical Sciences, Linköping University, Linköping, Sweden

**Keywords:** Adjuvant analgesics, Dysmenorrhea, Melatonin, Menstruation, Pelvic pain, RCT

## Abstract

**Purpose:**

Dysmenorrhea is a common, recurring, painful condition with a global prevalence of 71%. The treatment regime for dysmenorrhea includes hormonal therapies and NSAID, both of which are associated with side effects.

A dose of 10 mg melatonin daily has previously been shown to reduce the level of pelvic pain in women with endometriosis. We chose to investigate how this regime, administered during the week of menstruation, would affect women with dysmenorrhea but without any signs of endometriosis, as adjuvant analgesic treatment.

**Methods:**

Forty participants with severe dysmenorrhea were randomized to either melatonin or placebo, 20 in each group. Our primary outcome was pain measured with numeric rating scale (NRS); a difference of at least 1.3 units between the groups was considered clinically significant. Secondary outcomes were use of analgesics, as well as absenteeism and amount of bleeding. Mixed model was used for statistical analysis.

**Results:**

Eighteen participants completed the study in the placebo group and 19 in the melatonin group. Mean NRS in the placebo group was 2.45 and 3.18 in the melatonin group, which proved to be statistically, although not clinically significant.

**Conclusion:**

This randomized, double-blinded, placebo-controlled trial could not show that 10 mg of melatonin given orally at bedtime during the menstrual week had better analgesic effect on dysmenorrhea as compared with placebo. However, no adverse effects were observed.

**Clinical trials:**

NCT03782740 registered on 17 December 2018.

## Introduction

Dysmenorrhea is a common, recurring, painful condition with a global prevalence of 71% with a negative impact on academic performance [1] and is shown to disrupt cognitive performance [2]. The treatment regime for dysmenorrhea is non-steroid anti-inflammatory drugs (NSAID) and/or hormonal suppression [3]. Hormonal therapies and NSAIDs are both associated with side effects motivating the need for additional treatment options.

In primary dysmenorrhea, defined as painful menstruation without pelvic organ pathology, leukotrienes (LT) and prostaglandins (PG) are released from the uterus causing vasoconstriction and myometrial contractions, leading to hypercontractility of the uterus, ischemia, and pain [4]. The severity of symptoms are directly proportional to the type and amount of PG released into the systemic circulation during sloughing of the endometrial lining [5]. In addition, women with dysmenorrhea have a hyper-sensitization of pain fibers [6] and a high level of brain-derived neurotrophic factor (BDNF) in serum with a positive correlation to the intensity of dysmenorrhea [7], suggesting that primary dysmenorrhea has the characteristics of both acute and chronic pain.

Melatonin is a hormone regulating the circadian rhythm, synthesized, and secreted mainly from the pineal gland in the brain. The synthesis is synchronized with the light/dark cycle by photosensitive ganglion cells in the retina of the eye [8] and blocked by light at night. Secretion reaches peak levels at 02–04 am night [9]. Most of its metabolism occurs in the liver via cytochrome P450-mediated oxygenation, mainly by CYP1A2, and is excreted in urine [10].

A recent systematic review suggests that women with dysmenorrhea have higher levels of oxidative stress than healthy controls [11]. Melatonin has well reported anti-oxidative properties [12]. In addition to analgesic and anti-oxidative effects melatonin has proved to regulate contractions of the myometrium [13], suggesting several properties of high interest in treating dysmenorrhea.

The analgesic effect of exogen melatonin has been demonstrated in both acute and chronic pain, including inflicted, experimental pain [14], post-operative pain [15], fibromyalgia [16], irritable bowel syndrome [17], and cluster headache [18], with dosages ranging between 3 and 10 mg melatonin given orally. Ten-milligram melatonin ingested daily reduced dysmenorrhea in women with endometriosis and lowered the level of BDNF [19].

We chose to investigate how 10 mg melatonin daily, as an adjuvant analgesic during menstruation, would affect women with dysmenorrhea without signs of endometriosis.

## Materials and method

We conducted this randomized, double-blinded, placebo-controlled trial at Södersjukhuset, one of the largest hospitals in Stockholm, Sweden. Participants were recruited between March and December 2019. Prior to enrolment, a written informed consent was obtained from the participants. The trial was conducted in accordance with the principles expressed in the Declaration of Helsinki.

Call for participation was advertised on posters in the hospital, in gynecological outpatient clinics, in maternity care outpatient clinics, and on social media. Women aged 18–45 with regular menstruation, rating their dysmenorrhea 7 or higher on a numeric rating scale (NRS) during the most painful day, speaking and understanding Swedish, and in good general health were screened at the clinics Research Center for Womens’ Health at one of two doctors in charge of the trial. Screening visit included medical history, a pregnancy test, and vaginal ultrasound to identify those with manifest signs of endometriosis or other significant pathology for exclusion. Screening continued during one observational menstrual cycle, during which pain was recorded daily, and evaluated prior to inclusion. Exclusion criteria were smoking, pregnancy, prior or current liver or kidney disease, endometriosis, ongoing use of melatonin, alteration of any medication during the last 3 months, and use of opioids.

After inclusion, participants were randomized to 10 mg melatonin or placebo, each dose identical and dispersed in two capsules of 5 mg melatonin or placebo (both manufactured for the trial by APL, Stockholm, Sweden). The study drug was taken at bedtime daily for seven consecutive days with start on the first day of menstrual bleeding. Participants were instructed to continue with their usual pain medicating regime, if needed, for the 3 months of the study with no alterations. The duration of the study was one observational cycle followed by two interventional cycles, three menstrual cycles in total.

Our primary outcome was mean value of pain recorded daily during the week of menstrual bleeding. Secondary outcomes were use of analgesics, amount of bleeding, days of bleeding, days of pain, absenteeism, and potential effect on cognition.

Assessments were made daily with an online questionnaire sent via email to the participants. Registration started on the first day of menstrual bleeding. The worst pain of the day was recorded using the NRS, a scale from 0 to 10, where 10 is the worst imaginable pain. The use of analgesics was recorded daily with specification of number of tablets and dosage. Absenteeism was recorded daily. Bleeding was recorded daily through a pictorial blood loss assessment chart (PBAC). A reminder was sent by text message if needed. Cognition was assessed with a cognitive assessment software, CANTAB® (Cambridge Cognition 2019. All rights reserved. www.cantab.com). The cognition test battery, assessing motor screening tasks, reaction time, rapid visual processing, paired associates learning, and spatial working memory, was performed on a tablet computer at the Research Center for Women’s Health at Södersjukhuset, during the observational cycle and the last treatment cycle, respectively.

Daily recording of potential adverse effects was also made, and general experience of the study drug was evaluated at completion of the study.

Study data was collected and managed using REDCap electronic data capture tools (9.5.9 Vanderbuilt University, Nashville TN, USA) hosted at Karolinska Institutet.

To detect a clinically significant reduction of NRS of 1.3 units [20] with a power of 80% and a 2-sided alpha value of 0.05, 15 participants in each group were needed. We included 20 participants in each group, all in all 40 participants, to compensate for potential dropouts.

Participants were included consecutively upon a visit with the research nurse. Randomization was made by blocks of 4 by the manufacturer of the study drug, who provided consecutively numbered drug containers. The randomization key was retrieved and opened after the last participant had completed the study, thus assuring that the study blind was maintained during the treatment phase of the trial.

The main characteristics of the study population are presented in Table 1. In the placebo group two participants had anxiety, two had migraine, one had vestibulitis, and one had both hypothyroidism and depression. Fourteen subjects reported no co-morbidities. In the melatonin group two participants had premenstrual syndrome and one had celiac disease, hypothyroidism, a herniated disk, hyperthyroidism, polycystic ovary syndrome, and rosacea, respectively. Twelve subjects reported no co-morbidities.

**Table 1:** Main characteristics of the study population and observations during the 7-day baseline cycle

	**Placebo**		**Melatonin**	
	*n*	Mean (SD)	*n*	Mean (SD)
Age	20	28.45 (7.16)	20	26.95 (5.20)
Weight, in kg	20	72.80 (16.67)	19	68.84 (13.12)
Number of pregnancies	18	0.78 (1.22)	20	0.45 (1.0)
Pain, mean^a^	19	3.61 (.96)	20	4.35(1.71)
Days of pain	20	4.40 (1.18)	20	4.80 (1.70)
Total amount of analgesics in mg	20	4695.00 (3880.38)	20	4887.50 (5715.10)
Days of bleeding	20	4.80 (0.95)	20	5.35 (1.09)
Total PBAC	20	120.15 (59.72)	20	125.55 (122.20)
**Contraceptive method**	*n*		*n*	
Condom	5		8	
Hormonal IUD	2		1	
Cupper IUD	2		1	
COCP	0		2	
None	11		8	

^a^Pain refers to mean value of numeric rating scale (NRS) of the 7 days of observation. One participant in the placebo group failed to report the level of dysmenorrhea for every day in the baseline cycle

A mixed model was used to test the effect of the intervention and time. The inference was made on treatment cycle 1 and 2 excluding day 1, since the participants started the treatment on the evening of day 1. Three different covariance structures were tested by comparing −2 log-likelihood with chi-squared test on each outcome variable: unstructured, first-order autoregressive (AR (1)), and compound symmetry. The best fit was obtained with an unstructured model for outcome 1, with fixed effect and fixed intercepts. AR (1) showed the best fit for outcome 2, with fixed effects and random intercept. To test the effect of the two groups at each separate time point, an interaction effect between time and groups was tested in the selected model.

Unpaired *t*-tests were used to compare mean days of dysmenorrhea, days of bleeding, amount of bleeding, and cognition.

Acceptability was analysed with Fisher’s exact test.

SPSS version 26 (SPSS, Chicago, IL) was used for data analyses.

The Regional Ethical Review Board at Karolinska Institutet approved the trial (2017/1177–21/2) on 23 August 2017. Registration at Clinicaltrials.gov was made in December 2018 (NCT03782740). The first participant was enrolled on 4 March 2019 and last patient last visit occurred on 28 February 2020.

## Results

**Fig. 1:**
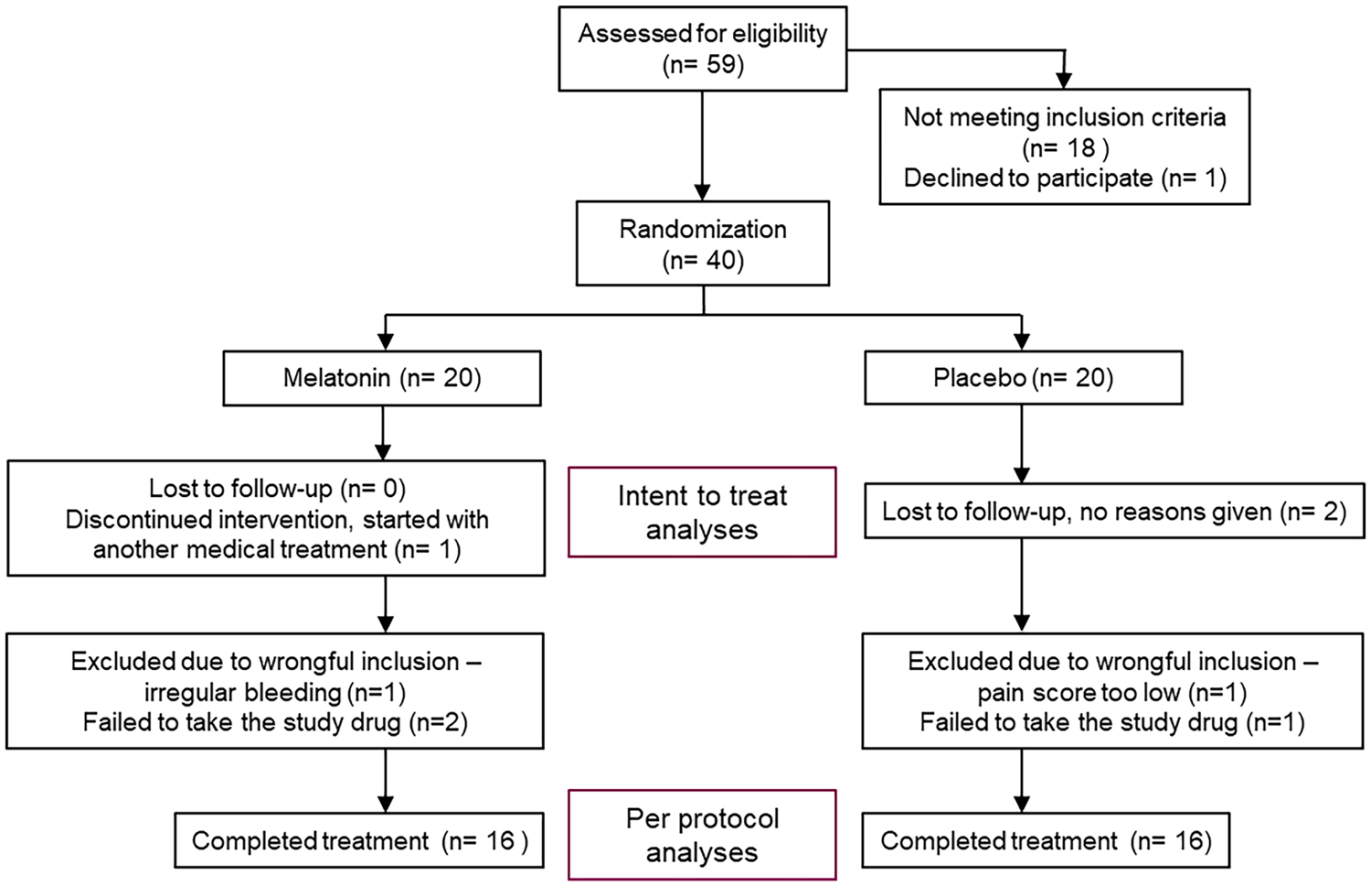
Flowchart showing recruitment and progress through the study. Intent-to-treat (ITT) analyses included 19 participants in the melatonin group and 18 in the placebo group. Per-protocol (PP) analyses were made on 16 participants in each group

Forty participants were randomized, 20 in each group, to either melatonin or placebo (Fig. 1). Clinical and demographic data of the two groups were similar (Table 1) with no statistically significant differences. No differences between the groups were seen in the tests assessing cognition (data not shown).

**Fig. 2:**
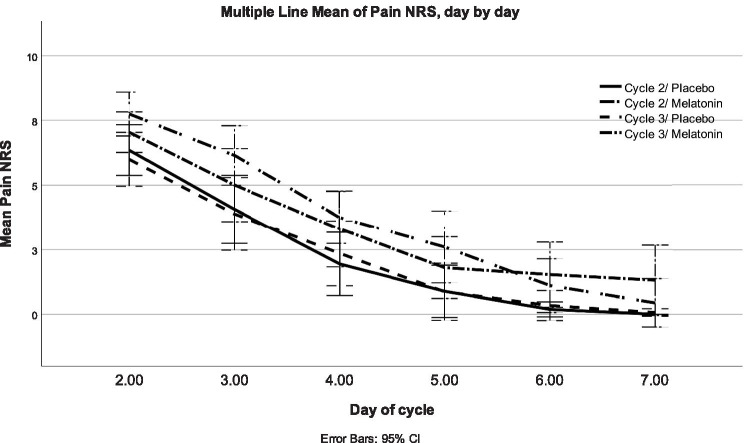
Dysmenorrhea day by day. Mean level of dysmenorrhea reflected by numeric rating scale (NRS) days 2–7 for the two study groups during the two treatment cycles, respectively

ITT analysis showed that the mean NRS of both treatment cycles was 0.73 units lower in the placebo group which proved to be statistically significant using mixed model analysis (Table 2). The difference of mean NRS between the study groups of the baseline cycle was 0.74 units, with no statistical significance using unpaired *t*-test. Mean NRS was lower in the placebo group in both treatment cycles (Fig. 2).

**Table 2:** Mean study outcomes of the two 7-day cycles (ITT)

**Outcomes**	**Treatment**	***n***	**Adjusted mean (SD)**	**Adjusted mean difference**	**95% confidence intervals**	***P*-value**
Pain, mean^a^	Placebo	18	2.45 (2.94)	−.73	−1.30 to −.16	.015
	Melatonin	19	3.18 (3.37)			
Amount of analgesics (mg)^a^	Placebo	18	464.032 (986.20)	−115.31	−497.64 to 267.02	.505
	Melatonin	19	579.342 (1192.00)			
Days with dysmenorrhea^b^	Placebo	18	3.89 (1.28)	−.53	−1,56-.49	.149
	Melatonin	19	4.42 (1.74)			
Days with bleeding^b^	Placebo	18	4.78 (.94)	−.17	−.93-.59	.329
	Melatonin	19	5.03 (1.15)			
PBAC — pictorial blood loss assessment chart^b^	Placebo	18	127.17 (48.66)	−29.78	−118.26-58.70	.246
	Melatonin	19	156.95 (178.48)			

^a^Analyzed with mixed model analysis

^b^Analyzed with unpaired *t*-test, cycle 3

Similar results were seen in the per-protocol analyses with the mean difference of 0.47 units, *P* = 0.09, CI −1.00 to −0.07 (mean 2.64, sd = 2.84 in the placebo group, and mean 3.10, sd = 3.36 in the melatonin group). Adjusting for weight did not affect the results.

No significant differences were seen between the groups regarding amount of bleeding (PBAC), use of analgesics, level of absenteeism, or in any of the cognition tests.

No adverse effects were reported. There were no observed differences regarding acceptability between the groups. Two participants used combined oral contraceptive pill (COCP), which has been shown to result in a higher level of serum melatonin compared with controls [21]. They were both randomized to the melatonin group and reported no adverse effects. Both reported a good experience with the study drug.

A majority in both groups wished to continue with the treatment, 61% in the placebo group and 63% in the melatonin group. Fifty-three percent in the melatonin group would recommend the treatment to a friend, 39% in the placebo group. No statistically significant differences in the acceptability of the study drugs were identified.

There were 20 days of missing data, 10 in each treatment group, all representing the last days of the cycle with no pain and no bleeding, suggesting no impact on the results.

## Discussion

Ten-milligram melatonin given during the menstrual week showed no clinically significant difference in the level of dysmenorrhea compared with placebo. We compared mean NRS for 7 days irrespectively of the number of days with dysmenorrhea, which gives seemingly low values of mean NRS considering most participants had fewer days of dysmenorrhea than 7.

No differences were observed in the secondary outcomes, use of analgesic drugs, number of days of pain, and bleeding, respectively, or the amount of bleeding. No adverse effects were reported, and no one reported daytime sleepiness after receiving melatonin treatment. Our assessment of cognition including reaction time and rapid visual processing showed no difference between the groups. In concordance with prior studies, tolerability and acceptance was good.

The study drug was ingested at bedtime to harmonize with the cyclicity of endogenous melatonin. For many participants the pain was already manifest when the treatment was initiated, on the evening of the first day of menstruation. This may have been too late to give a pain reduction. Considering that the time to maximal concentration (*t*_*max*_) of melatonin generally occurs around 50 min after ingestion, and also taking into account its short half-life in humans (20–40 min) [22], a prophylactic regime, given 45 min prior to pain onset, might have been favorable, but too challenging to administer due to the relatively unpredictable nature of dysmenorrhea. We did not evaluate plasma concentrations of melatonin, which would have provided valuable information, since bioavailability is low at 15% and is associated with high inter-subject variability [22].

If there is a correlation between pain regulation and melatonin, it should be possible to find an alteration in the endogenous melatonin concentration and the perception of pain. Nelson et al. [23] showed a decrease in melatonin concentration in saliva within 5 min after an acute pain stimulus. In addition, Almay et al. found lower levels of endogenous melatonin in serum and urine in patients with chronic pain [24]. Studies have shown conflicting results regarding a suggested cyclic alteration of melatonin levels following the menstrual cycle [25–27]. To our knowledge there are no studies measuring the endogenous levels of melatonin in women with dysmenorrhea.

Dose, administration route, and timing are of interest for future studies. Perhaps a prophylactic regime of melatonin administered with a mode of longer duration such as transdermal application, assessed with serum levels of melatonin as well as measuring its clinical effect on pain, could provide information on how to treat dysmenorrhea with melatonin.

The strengths of the study include the comparison of melatonin and placebo in a double-blinded, placebo-controlled trial; the low level of dropouts and missing data; and the low risk for recollection bias with a daily questionnaire. The study design offers high internal validity. We conducted this trial according to the CONSORT guidelines [28].

The study limitations involve study design as well as method. By including only self-selected women in good health, non-smokers with regular periods our study population is lacking in diversity with a possibly low external validity. The standard deviations and confidence intervals suggest a large variance which might suggest the study population being too small.

The first day is often the most painful, but due to not knowing the exact day the menstruation would commence we administered the first dose of melatonin in the evening of the first day of menstruation. Resulting, unfortunately, in that the first day of menstruation remained untreated.

## Conclusion

Our study could not show that 10 mg of melatonin given orally at bedtime during the menstrual week had better analgesic effect on dysmenorrhea as compared with placebo. However, no adverse effects were observed.

## Data Availability

All data are stored with REDCap, software.
